# Exploring Conformational Dynamics of the Extracellular *Venus flytrap* Domain of the GABA_B_ Receptor: A Path-Metadynamics Study

**DOI:** 10.1021/acs.jcim.0c00163

**Published:** 2020-04-01

**Authors:** Linn S. M. Evenseth, Riccardo Ocello, Mari Gabrielsen, Matteo Masetti, Maurizio Recanatini, Ingebrigt Sylte, Andrea Cavalli

**Affiliations:** † Molecular Pharmacology and Toxicology, Department of Medical Biology, Faculty of Health Sciences, UiT—The Arctic University of Norway, NO-9037Tromsø, Norway; ‡ Department of Pharmacy and Biotechnology, Alma Mater Studiorum—Università di Bologna, Via Belmeloro 6, I-40126 Bologna, Italy; § CompuNet, Istituto Italiano di Tecnologia, Via Morego 30, I-16163 Genova, Italy

## Abstract

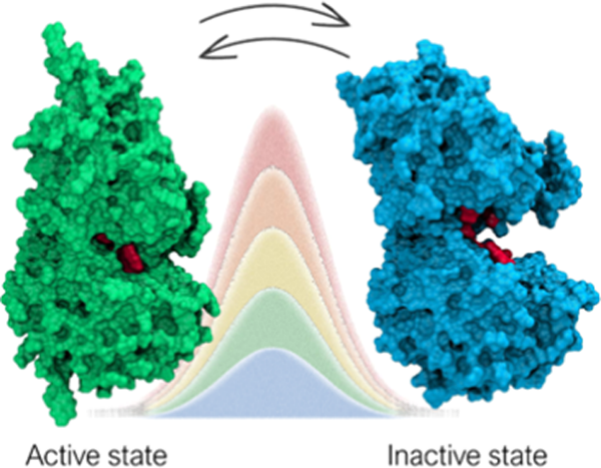

γ-Aminobutyric acid (GABA) is the main inhibitory neurotransmitter in the central nervous system (CNS). Dysfunctional GABAergic neurotransmission is associated with numerous neurological and neuropsychiatric disorders. The GABA_B_ receptor (GABA_B_-R) is a heterodimeric class C G protein-coupled receptor (GPCR) comprised of GABA_B1a/b_ and GABA_B2_ subunits. The orthosteric binding site for GABA is located in the extracellular *Venus flytrap* (VFT) domain of the GABA_B1a/b_. Knowledge about molecular mechanisms and druggable receptor conformations associated with activation is highly important to understand the receptor function and for rational drug design. Currently, the conformational changes of the receptor upon activation are not well described. On the basis of other class C members, the VFT is proposed to fluctuate between an open/inactive and closed/active state and one of these conformations is stabilized upon ligand binding. In the present study, we investigated the dynamics of the GABA_B1b_-R VFT in the apo form by combining unbiased molecular dynamics with path-metadynamics. Our simulations confirmed the open/inactive and closed/active state as the main conformations adopted by the receptor. Sizeable energy barriers were found between stable minima, suggesting a relatively slow interconversion. Previously undisclosed metastable states were also identified, which might hold potential for future drug discovery efforts.

## Introduction

γ-Aminobutyric acid (GABA) is the most abundant inhibitory neurotransmitter in the mammalian central nervous system (CNS) and central in modulating neuronal activity. GABA exerts its physiological effects through a distinct receptor system consisting of the ionotropic GABA_A_ and GABA_C_ receptors and the metabotropic GABA_B_ receptor (GABA_B_-R).^1^ Dysfunction in GABAergic and GABA_B_-R signaling is linked to a broad variety of neurological and neuropsychiatric disorders, including memory and learning deficits, addiction, epilepsy, schizophrenia, anxiety, and depression.^2−4^ The involvement of this receptor in human pathophysiology makes it a valuable drug target. A better understanding of the conformational dynamics associated with receptor activation is beneficial for new drug discovery.

**Figure 1:**
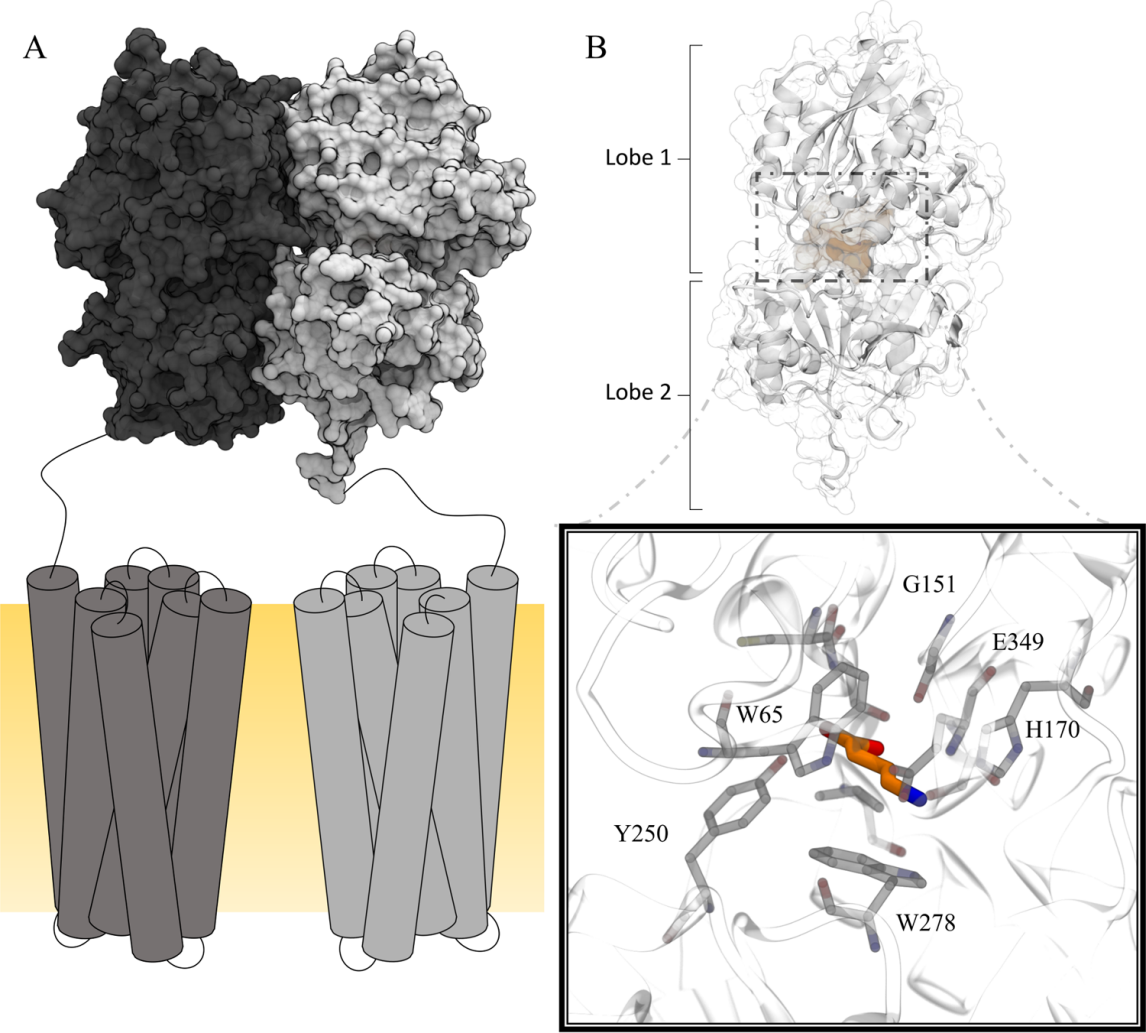
Schematic illustration of the GABA_B_-R. The heterodimeric GABA_B_-R is comprised of GABA_B1a/b_ (gray) and GABA_B2_ (black) (A). The 7TM domains are located in the membrane (yellow) (A). The orthosteric binding site is located in the extracellular VFT of GABA_B1a/b_ (B). Ligand binding is facilitated by interactions with key residues such as Tyr250 and Trp278 located in Lobe 2, Gly151, His170, and Glu349 located in Lobe 1 (black box, PDB ID: 4MS3 in complex with GABA).

GABA_B_-R is an obligate heterodimeric receptor comprised of GABAB_1a/b_ and GABA_B2_ subunits. The receptor belongs to class C of G-protein coupled receptors (GPCRs), together with the metabotropic glutamate receptors (mGlu1-8-R), the calcium-(CaSR), and sweet and umami taste receptors.^5^ Each subunit consists of an extracellular *Venus flytrap* (VFT) linked to a heptahelical transmembrane (7TM) domain (Figure 1),^5^ and hence GABA_B_-R does not contain the cysteine-rich linker that has been shown to play an important role in transmitting the activation signal from the VFT to the 7TM of other class C GPCRs.^5^ Likewise, the disulfide bridge that cross-links the VFT dimer of mGluRs is not present in the GABA_B_-R VFT.^5^ Radioligand binding studies and site-directed mutagenesis studies show that the orthosteric binding site of GABA_B_-R is located in the VFT of GABA_B1a/b_, while binding of ligands to the VFT of GABA_B2_ has not been observed.^6^ The 7TM domain of GABA_B2_ hosts an allosteric binding site and is responsible for G-protein coupling.^7,8^ GABA_B1a/b_ is dependent on dimerization with GABA_B2_ for trafficking from the endoplasmic reticulum (ER) to the cell surface as GABA_B2_ masks a retention signal present in the cytoplasmic tail of GABA_B1a/b_.^9^

Binding studies with recombinant receptor mutants, radioligand binding, and displacement assays have shown that the VFT of GABA_B1a/b_ is functional in absence of the GABA_B2_ VFT, although with reduced agonist affinities.^6,10−12^

The three-dimensional (3D) structure of the entire GABA_B_-R is not known; however, nine X-ray crystal structures of the VFTs cocrystallized with different agonists, antagonists, and one apo form have been published.^13^ The VFTs have a bilobular architecture where the two lobes (Lobe 1 and Lobe 2) are separated by a cleft and come into close contact upon agonist binding (yielding the active/closed state), hence the name VFT (Figure 1).^13^ Residues located in Lobe 1, such as Trp65, Ser130, G151, Ser153, His170, and Glu349, are responsible for anchoring ligands in the binding pocket and interacts interchangeably with both agonists and antagonists.^13^ Ligand interaction with the Lobe 2 residue Tyr250 is unique for agonists, and the Trp278 located in the same domain has been found to only interact with high-affinity antagonists in addition to agonists (Figure 1).^13,14^

The X-ray crystal structures of GABA_B_-R VFTs show that the GABA_B1a/b_ VFT is in a closed state in presence of agonists (the active/closed state) and in an open state when complexed with antagonists (the inactive/open state).^13^ However, mGluR VFT X-ray structures show that agonists and antagonists can both induce closed and open VFT conformations.^15^ Further, single-molecule Förster resonance energy transfer (smFRET) studies show that mGluRs in absence of ligand are in rapid exchange in the sub-milli-second timescale between active and inactive conformations, while binding of agonists is suggested to rapidly shift the equilibrium toward the active/closed state.^16^ The equilibrium between open and closed conformational states of mGluRs indicates that they are energetically equal, independent of ligand presence.^16^ Despite high sequence similarities between the eight mGluRs, kinetic differences between the receptors are identified.^17^ Although the sequence identity between the full mGluR2 and mGluR3 is as high as 70%, it has been shown that the active state of mGluR3 is more energetically stable than that of mGluR2 and that mGluR3 can be activated by Ca^2+^, while mGluR2 cannot.^17^ The sequence similarity between the mGluRs and GABA_B_-R VFTs is lower than between the mGluRs and is in the range of 43–48%.^18^

The activation mechanism of GABA_B_-R is partly elusive and mostly based on assumptions from knowledge regarding other class C members. In the present study, we aimed to investigate the structural dynamics of the GABA_B1_ VFT and describe the behavior of the system in the absence of ligands. To save computational time, only the monomeric form of the GABA_B1b_ VFT that contains the orthosteric site was considered as it has been demonstrated by multiple binding studies that GABA_B1b_ is functional without the GABA_B2_ VFT present.^11,12^ Six microsecond-long molecular dynamics (MD) simulations, using both the inactive/open and closed/active states of the GABA_B1b_ VFT as starting structures, were run to explore the functional dynamics of the receptor. The pool of trajectories obtained was instrumental to derive a suitable reaction coordinate that was exploited to guide the conformational transition through path-based enhanced sampling simulations.^19^

Enhanced sampling methods are well-accepted MD-based approaches suited for accelerating the occurrence of rare events and estimate the associated free energy surface (FES).^20^ A method that can be used is metadynamics,^20^ where a history-dependent biasing potential is added to selected degrees of freedom (also called collective variables (CVs)) to encourage the system to visit higher energy states. For the procedure to be effective and the reconstructed FES, accurate, a limited number of CVs must be able to fully characterize the process. Unfortunately, for complex phenomena like protein conformational rearrangements, the identification of a proper set of CVs is challenging^20^ and chemical intuition and/or trial and error procedures are required to fulfill this aim. However, when the start and endpoints of the transition and an educated guess of the underlying mechanism are available (i.e., the path bridging the end points), this step can be facilitated by using the so-called path-CVs (PCVs) formalism. When properly parameterized, PCVs may provide an optimal description of a transition process and in addition, PVCs also have the possibility of being iteratively improved.^21^ PCVs have successfully been used to study conformational transitions,^22,23^ ligand binding/unbinding,^24−26^ and ion conduction.^27^

In the present study, we use well-tempered metadynamics (WT-MetaD)^28^ combined with PCVs to fully characterize conformations underlying the transition between open and closed GABA_B1b_ VFT states. In particular, we show that open/inactive and closed/active VFT states are almost iso-energetic and separated by substantial energy barriers. Additionally, along the conformational transition, we identified metastable states that might play a significant role in the opening/closure mechanism and can be further exploited to drive structure-based drug discovery endeavors.

## Methods

### Protein Preparation

X-ray crystal structures of GABAB1b VFT in complex with antagonists (open/inactive state; PDB IDs: 4MR7, 4MR8, 4MR8), the agonists GABA and baclofen (closed/active state; PDB IDs: 4MS3, 4MS4, respectively), and one in apo form (open/inactive state; PDB ID: 4MQE) were selected for the study. The ligands and the GABA_B2_ VFT were removed from each X-ray crystal structure, and the remaining GABA_B1_ VFT structures were preprocessed in Schrödinger Protein Preparation wizard using default settings (hydrogens were added according to the physiological protonation states at pH value of 7; bond orders were assigned and disulfide bonds created).^29^ A minimization run was performed with converging heavy atoms at root mean square deviation (RMSD) of 0.3 Å.^30^

### Unbiased Molecular Dynamic Simulations

For each of the six processed GABA_B1_ VFT structures, a 1 μs long MD simulation was performed using GROMACS 2016 MD package^31^ and the AMBER99SB-ILDN force field.^32^ To set up the individual systems, *N*-methyl amide (NME) and acetyl (ACE) caps were added to the N- and C-termini, the protein was solvated in a cubical box adopting the transferable intermolecular potential 3P (TIP3P) water model,^33^ and the total charge of each system was neutralized by adding three Na^+^ ions. Energy minimization using the steepest descent minimization algorithm was performed and run until the maximum force of the system reached <1000 kJ/(mol·nm) using GROMACS 2016 MD package.^31^

System equilibration was achieved by performing a 100 ps MD simulation in the NVT ensemble followed by a 5 ns constant number of atoms, pressure, and temperature (NPT) equilibration, using the leap-frog integrator with a time step of 2 fs. The temperature was coupled to the stochastic v-rescale modified Berendsen thermostat^34^ at the target temperature of 300 K with a time constant of 0.1 ps. In the isothermal–isobaric ensemble, the pressure was controlled with the Parinello–Rahman barostat^35,36^ with a coupling constant of 1 ps and a reference pressure of 1 bar. All bonds involving hydrogen atoms were constrained with the LINCS algorithm.^37^ The Verlet cutoff scheme was used with short-range electrostatic and van der Waals cutoff at 14 Å. The long-range electrostatic interactions were treated using the particle mesh Ewald (PME) method with a 4th-order spline and Fourier spacing of 1.6 Å. Following system equilibration, production runs of 1 μs were performed as an extension of the previously described NPT ensemble and by saving conformations every 10 ps.

MD simulations were monitored by calculating the root mean square deviation (RMSD) and root mean square fluctuation (RMSF) over Cα atoms using a closed/active crystal structure as a reference (PDB ID: 4MS3). The RMSF analysis showed that residues in Lobe 2 fluctuated less than residues in the Lobe 1 and selected Cα atoms of this lobe were therefore used in all subsequent structure alignments (see Table S1 and Figure S1).

### Optimization of the Path Variables

Transitions between open/inactive and closed/active conformational states of the GABA_B1_ VFT were characterized by well-tempered metadynamics (WT-MetaD) and path collective variables (PCVs). PCVs provide an optimal description of the process under investigation provided that the end points of the transition are known and that an educated guess of the underlying mechanism is established. Specifically, with PCVs, the path joining the end points is described by an ensemble of intermediate structures in configurational space (*x*) that represent the so-called frame set (*i* = 1, 2, ..., *N*). Then, the progression along the path and the distance from it is evaluated through the following variables, respectively^38^
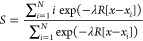
1
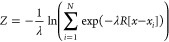
2In the definition of *S* and *Z*, λ is a tunable parameter controlling the smoothness of the mapping from the discrete frameset to the continuous space of the variables (see below). The distance of the current configuration from all members of the frameset is usually evaluated through the mean squared displacement (MSD) calculated between a predefined subset of atoms after optimal body superposition. Specifically, in eqs 1 and 2, *R*[*x* – *x*_*i*_] represents the MSD. The variables *x*_*i*_ are the Cartesian coordinates of the subset of Cα atoms belonging to Lobe 1 employed to evaluate the conformational transition after optimal alignment on the subset of atoms belonging to Lobe 2 that is acting as a reference frame (see Table S1 and Figure S1). Conversely, the *x* variables represent the current configuration of Cα atoms belonging to Lobe 1 during the metadynamics simulation. In this way, all sampled configurations are continuously and smoothly mapped onto the *S* and *Z* space. In this work, both the end points and the educated guess path used to parameterize PCVs were extracted from the MD trajectories obtained by the previous step.

**Figure 2:**
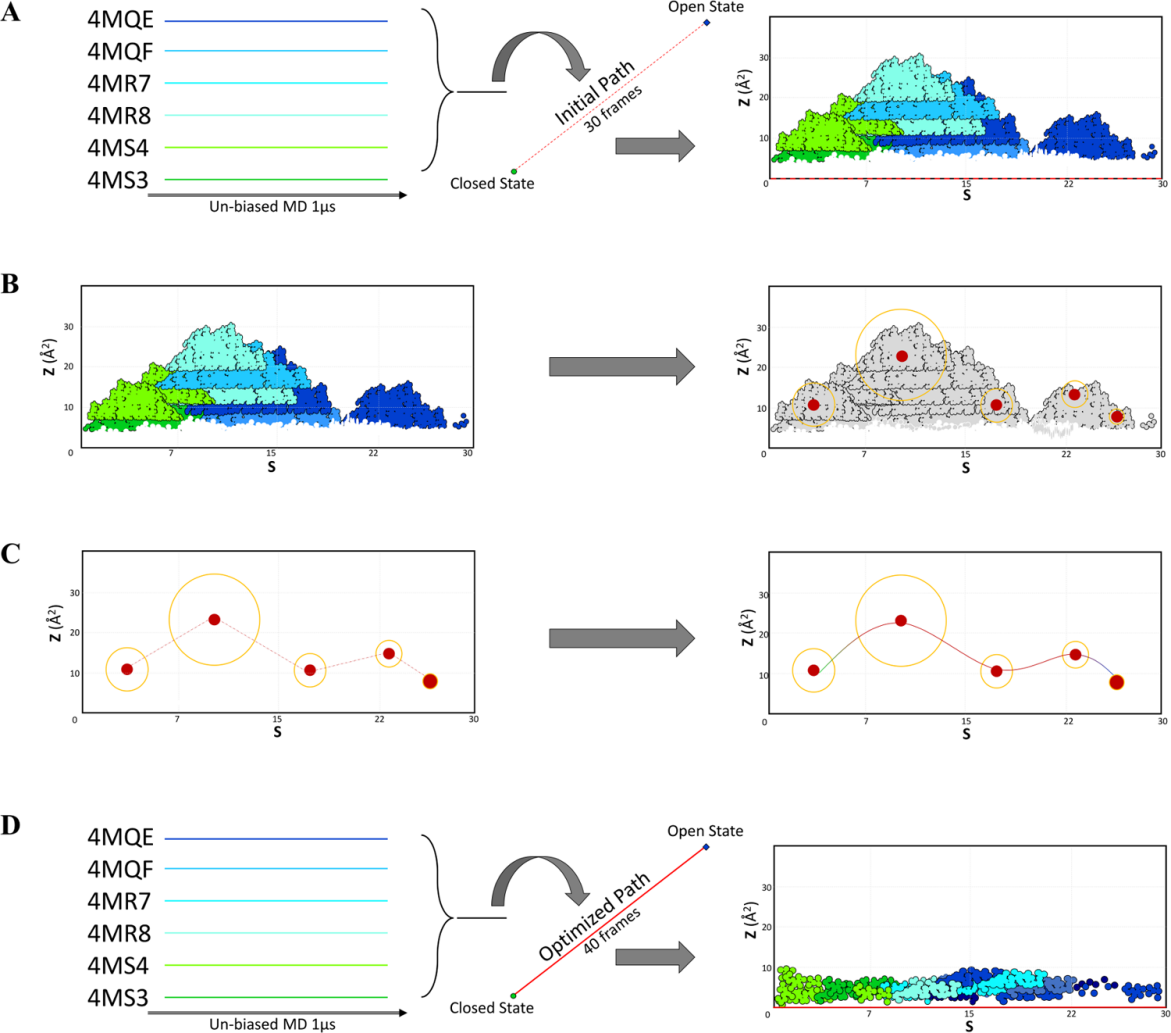
Overview of the main steps performed to obtain a path describing the conformation transition from the closed to the open state. (A) unbiased-MD projection of trajectories onto the initial path, (B) cluster analysis for identification of physically meaningful intermediate structures, (C) bridging the clusters to obtain the refined path, and (D) PCV validation through projection of the unbiased-MD trajectories onto the refined path.

**Figure 3:**
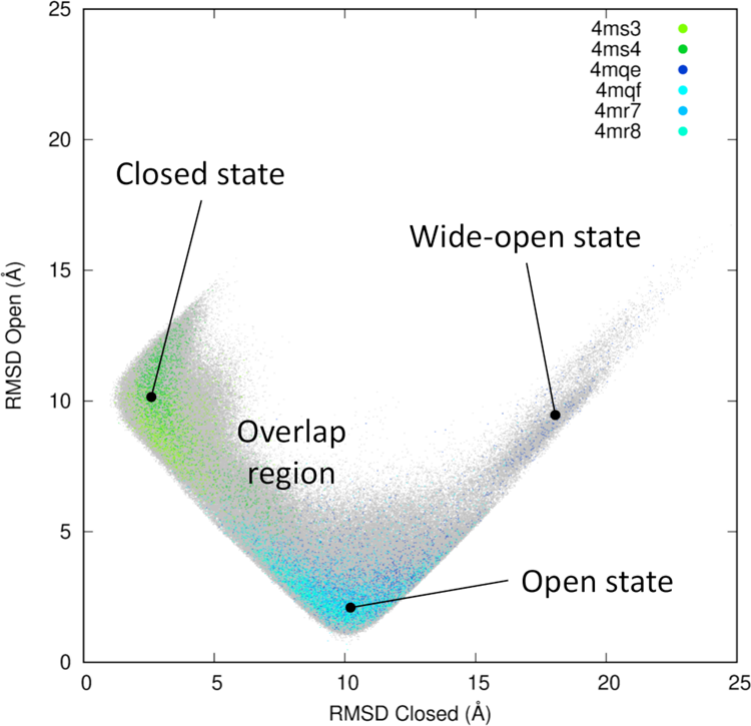
Projection of the six 1 μs long trajectories onto the two-dimensional (2D)-RMSD space. The profile is calculated using PDB ID 4MS4 and 4MQE as references for the closed and open states, respectively. The entire set of data points is shown as gray dots and the projection of individual trajectories are plotted with a greater stride. The color code is consistent with the legend inside the plot and used throughout this work (green for the closed state and blue for the open state).

The end points corresponded to the equilibrated conformations obtained from PDB ID: 4MS3 and PDB ID: 4MQE for the closed/active (*i* = 1) and open/inactive states (*i* = *N*), respectively. Notably, while the equilibrated structure of the closed/active state closely resembled the corresponding crystallographic geometry, the other end point represented a conformational state of GABA_B1_ VFT with a greater separation between the lobes as compared with the antagonist bound structures and the apo form. This state (hereafter referred to as “wide open”, see also Figure 3) could only be identified through MD simulations and was intentionally employed as an end point in the PCV parameterization to ensure that the entire conformational transition was covered. The remaining (*N* – 2) intermediate structures of the frame set were extracted from the aggregated trajectories in an iterative fashion. Each time a new guess path was generated, the size of the frameset (*N*) and the interframe distance could change, and therefore the value of λ needed to be modified accordingly. Here, λ was adapted based on the average MSD calculated between adjacent frames following the rule of thumb: λ = ln⟨|*x_i_* – *x*_*i*+1_|^2^⟩^–1^. The initial guess path was obtained through the interpolation scheme implemented in the Climber program^39^ using the structural information of the end points only. In contrast to other morphing tools, Climber does not interpolate conformations linearly but instead uses the restraining energy in a linear manner depending on the distance deviation between the current and the reference structure.^39^ This concept allows larger structural flexibility and permits the protein to be sampled around high-energy barriers. Each Climber step was followed by energy minimization of the predicted structure.^39^ The number of intermediates was set to a minimum of 150 minimized structures. Then, a total number of 30 equispaced frames were extracted with an in-house script and the λ value was set to 312 nm^–2^. In the *S*/*Z* framework, the total number of frames (*N*) must be chosen as a compromise between the accuracy of the mechanistic description (*N* large) and the computational convenience (*N* low). In this work, we tuned the frame set in a way to satisfy an average interframe distance of about 0.86 Å. As the quality of this path was evaluated to be a suboptimal representation of the true mechanism of the conformational transition (Figure 2A), we aimed to extract the guess path from the pool of available trajectories. We then performed a cluster analysis using the GROMACS clustering tool^31^ with an RMSD distance calculated from the Cα atoms with a 1.5 Å cutoff. The centroid conformation from each cluster was chosen as cluster representatives with specific *S* and *Z* values (see Table 1). The centroids corresponding to the most populated clusters (first 10 out of 35) were then selected and connected using Climber.^39^

**Table 1:** Number of Conformations Found in the Most Populated Clusters (Populations), and Corresponding Cluster-IDs^a^

cluster-ID	population	*S*	*Z* (Å^2^)
1	17 018	11.8	19.4
2	4960	2.6	3.2
3	3579	1	4.6
4	1140	8.6	6.6
5	1099	21.5	8.1
6	837	10.2	11.7
7	357	16.1	20.1
8	173	5.7	4.6
9	159	26.9	3.9
10	157	14.6	26.1
11	124	13.3	16.7
12	87	15.4	15
13	62	8	4.5
14	36	3	6.9
15	35	1	5
16	29	11.6	7.6
17	26	7.5	7.6
18	26	13.9	41.5
19	21	12.8	15.1
20	15	4.8	14.1

a Cluster centroids were selected, and *S* and *Z* values of these conformations were calculated (last two columns). Only the most populated clusters are shown.

The optimized path was then obtained by concatenating all disconnected partial paths obtained through Climber (for a total number of 1045 minimized structures) and selecting an ensemble of equispaced conformations in a way to obtain a frame set with *N* = 40, λ = 193 nm^–2^, and an interframe distance of 1.1 Å. Projection of the unbiased trajectories onto the newly optimized PCV space showed that all sampled points were now lying in the close proximity of *Z* = 0, indicating that a satisfactory representation of the transition was obtained. The schematic representation of the entire procedure is shown in Figure 2. The refined path was then used as a collective variable in a WT-MetaD simulation using GROMACS 2016^31^ patched with PLUMED.^40^ The temperature was set to 300 K and Gaussian hills were added in a regular interval of 1 ps with a height of 0.1 kcal/mol and the width of 0.2 for both variables, *S* and *Z*. The bias factor used to rescale the Gaussian height in the simulation was set to 8, and the total run length of the simulation was 2 μs. The free energy was reconstructed at the end of 2 μs of sampling and right after the main recrossing event at about 1.2 μs (see Figure S2A). Since after the recrossing event the system was found to be stuck in the closed/active basin, to avoid overfilling, we consider the FES obtained at 1.2 μs as the conclusive result of this simulation. For the sake of comparison, the one-dimensional (1D)-free-energy profiles and the 2D-FES obtained at the end of the 2 μs sampling are shown in Figures S2B and S3, respectively.

A second cluster analysis of conformations sampled during metadynamics was performed with emphasis on the key residues in the binding pocket as previously described, with an RMSD distance calculated from the heavy atoms of each residue using 1.5 Å as a cutoff. The conformations sampled only in three selected stationary points (selected in correspondence with the *S* value) were selected.

All data and PLUMED input files required to reproduce the results reported in this paper are available on PLUMEDNEST (www.plumed-nest.org), the public repository of the PLUMED consortium,^41^ as plumID:20.002.

### Committor Analysis

The quality of the *S* variable in describing the conformational transition was assessed through a committor analysis.^42^ Specifically, this analysis quantifies the commitment probability of trajectories initiated from a given point in the CV space to reach one of the main metastable states (in this context, usually referred to as state A and state B). Here, state A (corresponding to the closed state) was defined as the region spanned by *S* ≤ 11.5 and *Z* ≤ 0.05 nm^2^, while state B (corresponding to the open state) was defined as *S* ≥ 21.5 and *Z* ≤ 0.05 nm^2^. Hundred unbiased MD runs were started in close proximity of the transition state at *S* = 16.5 and *Z* = 0.05 nm^2^ and were automatically terminated once the A or B state was reached through the PLUMED software.^40^

## Results

### Unbiased MD Simulations

The Cα RMSD of each trajectory (Figure S4) was calculated using an X-ray crystal structure in the closed/active state (PDB ID: 4MS4) as a reference structure. The results showed that the Cα RMSDs from the unbiased MD simulations of GABA_B1b_ VFT in the closed/active state (PDB IDs: 4MS3 and 4MS4) diverged at the beginning of the simulation (first 250 ns), but afterward their RMSDs reached similar values, indicating an overlapping conformational space for the rest of the simulation (Figure S4, green plots). Cα RMSD plots obtained from the simulation of GABA_B1b_ VFT in inactive/open states (PDB IDs: 4MR7, 4MR8, 4MQE, and 4MQF) showed similar trends, except that the RMSD of the apo structure (PDB ID: 4MQE) increased considerably during the last part of the simulation (>850 ns, Figure S4, dark blue plot), reflecting that the VFT at this stage of the dynamics adopted a wide-open state. This behavior is highlighted by the 2D-RMSD plot (Figure 3), where a clear overlapping region of conformational states can be found, as well as the wide-open conformation occasionally reached by the simulations started from the apo structure. We note that the same subset of atoms used in the definition of the *S* and *Z* variables was used to obtain these plots.

Importantly, a complete conformational transition could not be observed through this initial set of simulations. In spite of this, the obtained pool of trajectories was sufficient to derive a suitable reaction coordinate that was then exploited to guide the conformational transition through path-based enhanced sampling simulations.^19^

### Metadynamics Simulations

To evaluate the dynamical behavior of GABA_B1b_ VFT domain, a guess path describing the transition of the VFT from the active/closed to inactive/open state was optimized using the information coming from the six 1 μs long trajectories of unbiased MD simulations. The initial path was generated by using the Climber morphing tool.^39^ Evaluation of this path by projecting the six MD trajectories on the space spanned by PCVs (Figure 2A) showed that most of the points representing individual configurations sampled by unbiased MD were far away from the guess path, ideally located at *Z* = 0 for all values of *S*. This indicated that the initial path was a poor representation of the conformational transition mechanism. Despite no full conformational transition obtained, the individual trajectories were partly overlapping in the PCV space (Figure 3) and we therefore aimed to exploit the information in the pool of trajectories to refine the path and possibly obtain a more trustworthy representation of the mechanism. By selecting conformations from the MD clusters, we ensured that the new path not only covered the whole transition but also retained salient features of the mechanism as captured by the most populated conformations sampled along the pathway. The projection of the MD trajectories on the new PCV space showed low *Z* (<3 Å^2^) values, indicating that the final obtained path was a reliable representation of the transition (Figure 2D).

**Figure 4:**
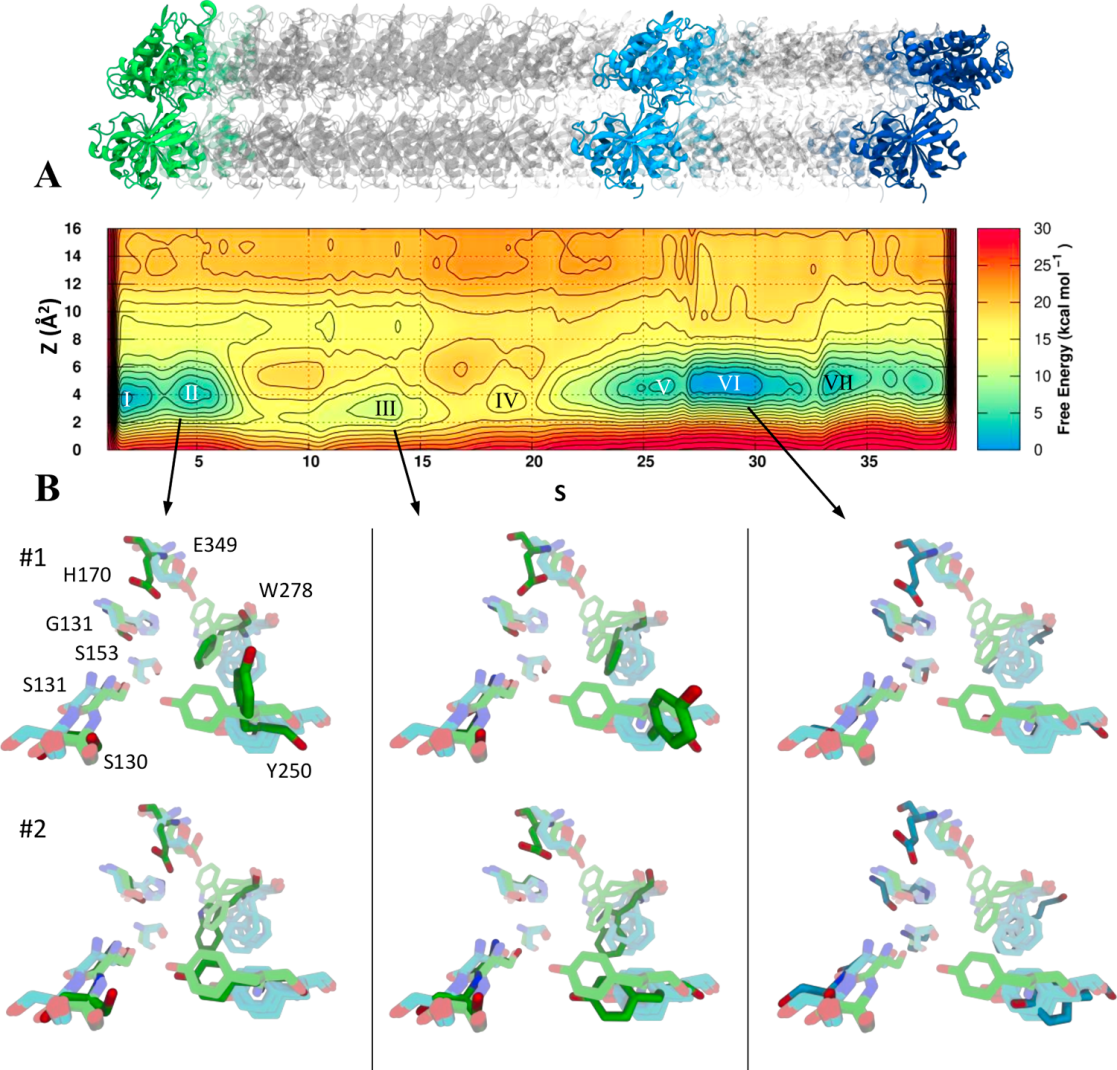
Closed-to-open conformational transition of the GABA_B1b_ VFT domain. (A) FES obtained from 1.2 μs long metadynamics simulation as a function of the *S* and *Z* variables. The contours are plotted every 2 kcal/mol. The bottom panel (B) shows the orientation of the key residues implicated in ligand binding. Pastel green and cyan residues represent the X-ray crystal conformations of the binding site in the closed and open state, respectively, whereas the dark green and blue residues represent the adopted conformation of the same residues in three major minima (I, II, VI). The two main clusters of each minimum are reported (#1, #2).

The FES obtained after 1.2 μs of metadynamics simulations showed a local minimum in the first frames of the path, corresponding to the closed (active) conformation (basins I and II, Figure 4A). A second broad minimum covering the frames close to the intermediate part of the path corresponded to the inactive (open) conformation (basins V and VI, Figure 4A). These two regions were almost iso-energetic with a slight preference toward the open state and were separated by energy barriers of approximately 16 kcal/mol. The committor analysis showed that the *S* variable was reasonably well-suited to describe the transition under investigation, especially considering the complexity of the domain motion and the number of atoms involved. Starting a series of unbiased MD simulations nearby the transition state gave an estimated probability to reach the open conformation of 0.31 (Figure S5). The orientation of amino acids involved in the binding of agonists and antagonists in X-ray crystal structures was compared to their orientation obtained from metadynamics simulations (Figure 4B). Cluster analysis performed for the two main metastable states (II and VI) gave insight into conformational changes of the binding site amino acids along the transition. In particular, the conformational changes of Trp278 and Tyr250 relative to the X-ray complexes were analyzed in detail. In the second main metastable basin corresponding to the stable closed conformation (basin II, Figure 4A), Trp278 showed a slight outward rotation compared with the crystal structures cocrystallized with the agonist GABA and the agonist baclofen (#1 in Figure 4B). Also, Tyr250 adopted a different orientation (outward rotation) compared with the crystal structures in complex with agonists. The rotation of Tyr250 was also seen in other metastable states (basin III, Figure 4A) that corresponded to intermediate conformations observed during the closed-to-open movement. Analyzing the same residues in basin VI, corresponding to the open state (Figure 4A, basin VI), we notice that Trp278 goes through a rotational movement causing the residue to occupy the same space as seen in the inactive/open X-ray crystal structure (#1, Figure 4B), while Tyr250 rotated back to the original orientation observed in the active/closed X-ray structures (#1, Figure 4B). Other residues playing key roles in ligand binding (His70, Gly151, Ser153, Ser130, and Ser131) maintained their geometry during the simulation, showing high structural stability even in the absence of ligands. Glu349 represented the only exception to this behavior, possibly due to the absence of a positively charged counterpart.

## Discussion

In the present study, the conformational dynamics of the GABA_B1_ VFT was studied through MD simulations initiated from six distinct X-ray crystal structures of the GABA_B1_ VFT. Four of the selected structures represented the inactive/open conformation, while two VFT represented the active/closed state. On the basis of the sampled conformations, a path was generated describing the full transition from the closed to the open conformation. The path was then used as a CV in a subsequent metadynamics simulation to resample the conformational movement and estimate the associated FES.

Many biologically important events such as protein–protein interactions and large domain motion occur in the millisecond (ms) to second timescale and major conformations are often separated by high energy barriers.^43^ In spite of recent improvements in hardware and software,^44^ interesting conformational events of macromolecules are still inaccessible for unbiased sampling methods due to the limitation of the timescales (>ms). For example, the rearrangements of the transmembrane domain of mGluR1 during receptor activation are in the 20 ms timescale.^45^

The analysis performed on the aggregated trajectory obtained from the six unbiased MD simulations showed that a viable reaction coordinate could be extracted to fully describe the conformational transition of the GABA_B1_ VFT, even though individual trajectories spanned only a limited portion of the conformational space. The analysis also showed that one crystal structure initially in an inactive/open conformation (PDB ID: 4MQE), sampled a previously undescribed wide-open state during the last part of the simulation. This might be a conformation less frequently accessed by the receptor, even though we cannot rule out that the absence of the GABA_B2_ VFT might have exacerbated this behavior. Future investigations are needed to clarify this aspect. However, we included this conformation as an endpoint of our optimized path to ensure that the entire transition could be sampled during the following metadynamics simulation.

Metadynamics is a powerful technique for accelerating rare events and reconstructing the free energy associated with selected movements such as conformational changes and binding of ligands.^20,24,27^ With metadynamics, the sampling is accelerated by adding a bias potential to a few CVs describing the event one aims to investigate. This allows the system under investigation to efficiently cross energy barriers and thereby explore “infrequent events” that are only occasionally observed or even inaccessible by conventional MD simulations.^46^ Specifically, in a metadynamics simulation, a history-dependent bias potential is added as a sum of Gaussians deposited at a regular time along the CV space.^46^ As Gaussians are deposited, the underlying bias potential grows and encourages the system to explore new regions of the CV space by crossing saddle points and thus reaching previously unexplored low-energy basins. Additionally, the bias potential can be used to estimate the underlying free energy as a function of the CVs.^46^

Identification of reliable CV depends on knowledge of the target under investigation and is very important for the reliability of the simulation. Among popular CVs, atomic distances, dihedrals, angles, and atomic contacts can be mentioned.^20^ Selecting appropriate CVs is highly challenging as macromolecules have a huge number of degrees of freedom and topological complexity. A CV must describe the slow motion of the system, if this motion is relevant for the process under investigation.^20^ Also, the chosen CV should be able to distinguish between the initial and final states, including relevant intermediates.^46^ As an example, Branduardi et al. applied metadynamics to describe the translocation of tetramethylammonium (TMA) in the acetylcholinesterase (AChE) gorge using the distance between the TMA and AChE as a single CV.^47^ The results showed that an important slow motion was neglected, resulting in a fluctuating behavior and preventing a proper convergence of the FES.^47^ An aromatic residue was blocking the gorge, and additional CVs describing this rare event was necessary for obtaining the correct FES.^47^ To avoid such a trial and error procedure, we adopted a path-CV formalism. PCVs are flexible descriptors allowing competition of the progression along a user-defined path and the distance from it, thereby reducing the problem of finding a limited number of correct CVs for describing a complex movement. The equilibrated state corresponding to the active/closed state was selected as the initial structure for the path, while the endpoint was represented by the wide-open conformation sampled in the unbiased MD simulation. Conversely, the intermediate frames consisted of an ensemble of conformations generated from interpolation and extraction from the pool of trajectories obtained through unbiased MD (see the Methods section). We acknowledge that more elegant solutions based on Markov State Models are currently available to extract reaction coordinates from previous MD simulations.^26,48^ However, these approaches require much more thorough sampling than what could be achieved in the present work. Moreover, we note that during metadynamics, the system is allowed to explore regions in configurational space significantly distant from the input path. From this standpoint, PCVs should be regarded as a nonlocal reaction coordinate.

The X-ray crystal structures are assumed to be in a low-energy conformation and should therefore correspond to two local minima on a FES. The FES obtained by using our path showed local energy minima at values of the *S* variable corresponding to the closed/active X-ray structure (basins I and II in Figure 2A) and the open/inactive receptor conformation (basins V and VI), with equivalent low *Z* values and similar energy. These major conformational states turned out to be separated by substantial energy barriers. Other metastable states were observed at relatively high-energy regions of the PCV space (basins III and IV in Figure 2A). We suggest that these intermediate conformations are effectively populated only upon ligand binding. The wide-open state was also included in definition of the optimized path to probe its stability. Despite this conformation being actually sampled during metadynamics, the less favorable energetics supports the theory that this conformation is only occasionally visited.

The energy barrier separating the two local minima corresponding to the open and closed states was estimated to be in the order of 16 kcal/mol. The height of this barrier also supports that GABA_B1_ VFT requires an agonist to undergo efficient receptor closure, unlike mGluRs that oscillate between the two states independent of ligand binding as described by Olofsson et al. and Grushevskyi et al. using mGluR2 and mGluR1, respectively.^16,45^ The analysis of the key residues participating in ligand binding showed that the geometry was unchanged for most of the residues during the simulation, also supporting the need for an agonist to induce conformational changes. We wish to underline that none of these residues were directly influenced by the simulation bias, which only has the purpose of accelerating the domain motion between the open and closed states.

It is important to be aware of possible methodological limitations that might have played a role in determining the high energetic barrier recorded. We note that some of these limitations are related to widely accepted simulative setups, while others are more specifically inherent to the current investigation. As previously mentioned, for computational convenience, only the monomeric form of the GABA_B1b_ VFT was considered in the simulations. Although experimental studies show that the monomer is functional with lower agonist affinity in the absence of GABA_B2_ VFT^11,12^ and that the conformation of GABA_B2_ is nearly independent of the presence of GABA_B1a/b_,^13^ we cannot rule out that potential dimeric cooperative effects might be neglected in our simulations. From this standpoint, the simulations presented in this work can be regarded as a control for future investigations on the energetics of the dimer. Another possible reason for deviations from experimental studies performed on other class C members can be related to the fact that the simulations were performed including only the minimal amount of ions required to neutralize the overall charge of the system in the simulation box. In other words, the physiological ionic strength was not explicitly taken into account. While this is quite a popular simulative setup, it is difficult to assess any possible implications of this choice in quantitative estimates such as free-energy barriers.

Concerning the reliability of the mechanistic interpretation, there are two aspects worth considering. The first one is related to the quality of the reaction coordinate, namely, the *S* variable. While the committor analysis revealed that the location of the transition state ensemble was reasonable, we note that the closet-to-open motion (or vice versa) was never sampled through unbiased MD simulations and a suitable procedure involving interpolation among cluster representatives was necessary to reconstruct the whole transition. Even though the usage of the *Z* variable lends nonlocality to metadynamics sampling, we cannot exclude that other competing routes might be involved in the domain motion. Since the optimized path was solely derived by information coming from prior unbiased MD simulations, these hypothetical routes might be hidden to our PCV space simply because of the limitation of sampling. This leads us to the second topic, which is related to the accuracy of the reconstructed free-energy profile by metadynamics. The convergence of results is a long-standing issue of metadynamics simulations. In this work, we used WT-MetaD that by virtue of the decreasing heights of the Gaussians is expected to converge the free energy to stationary values in the limit of exhaustive sampling. Unfortunately, the system might become trapped in some regions of the CVs space leading to an undesired oversampling and resulting in an unbalanced reconstruction of the FES. To avoid this behavior, it is a common practice to stop the metadynamics simulation right after the achievement of the main recrossing event. In this work, the metadynamics simulation was carried out for a total simulation time of 2 μs and the free energy reconstructed at 1.2 μs was considered to provide a faithful intepreteation of the process under investigation. Even though the presented results make us confident about the reliability of the relative energy difference among the main basins, it is possible that much longer (and possibly repeated) simulations would be required to properly converge the free energy at the transition state region.

## Conclusions and Future Perspectives

In this study, we have investigated the dynamics of the GABA_B1b_-R VFT in the absence of ligands by combining unbiased molecular dynamics (MD) with path-metadynamics. The results confirm that the relaxed open state and active closed state are the two main conformational states of the GABA_B1b_-R VFT. The two states are iso-energetic but separated by a substantial energy barrier. This result, together with the stable geometry of key residues in lobe 1 of the orthosteric binding pocket, indicates that the GABA_B1b_-R VFT will not oscillate between the two conformations in absence of a ligand contrary to mGluRs. This also explains the consistency of the crystal structures where all agonist- or antagonist-complexed conformations are open or closed, respectively. In a future perspective, a simulation using the dimer could also be beneficial to investigate the role of GABA_B2_-R on ligand binding, interface interactions and potentially the wide-open state observed in this study.

## Supporting Information Available

Atoms used to construct the path variables (Table S1 and Figure S1); time evolution of *S* during metadynamics and 1D-free energy profiles (Figure S2); 2D-FES at the end of the metadynamics simulation (Figure S3); RMSD evaluated during unbiased-MD simulations (Figure S4); committor analysis (Figure S5) (PDF)
