# Supplementary material for: Exploring Conformational Dynamics of the Extracellular Venus flytrap Domain of the GABAB Receptor: A Path-Metadynamics Study
Source: J Chem Inf Model. 2020 Apr 1;60(4):2294–303. doi: 10.1021/acs.jcim.0c00163 (PMC7997371; doi:10.1021/acs.jcim.0c00163)
Supplement: Supplementary file 1 — ci0c00163_si_001.pdf [file ci0c00163_si_001.pdf]

Supporting information

Exploring Conformational Dynamics of the Extracellular Venus Flytrap Domain of the GABA$_B$ Receptor: a Path-Metadynamics Study

Linn S.M. Evenseth,[*,†,#] *Riccardo Ocello,*[‡,§,#] *Mari Gabrielsen,*[†] *Matteo Masetti,*[*,‡] *Maurizio Recanatini,*[‡] *Ingebrigt Sylte,*[†] *and Andrea Cavalli*[‡,§]

[†]Molecular Pharmacology and Toxicology, Department of Medical Biology, Faculty of Health Sciences, UiT – The Arctic University of Norway, NO-9037 Tromsø, Norway

[‡]Department of Pharmacy and Biotechnology, Alma Mater Studiorum - Università di Bologna, Via Belmeloro 6, Bologna, I-40126, Italy

[§]CompuNet, Istituto Italiano di Tecnologia, Via Morego 30, I-16163, Genova, Italy

[#]These authors contributed equally to this work.

[*]Co-corresponding authors

Table S1. Cα atoms of amino acid residues were used for alignment (Lobe 2) and measurement (Lobe 1) during simulations of the $GABA_{B1b}$ VFT. The residue numbering is consistent with the numbering in the available X-ray crystal structures.

Lobe 1: Measurement								
**Residue**	Arg51	Ala52	Val53	Tyr54	Ile55	Gly56	Ala57	Leu58
	Phe59	Gln69	Ala70	Cus71	Gln72	Pro73	Ala74	Val75
	Glu76	Met77	Ala78	Leu79	Glu80	Asp81	Val82	Asn83
	Tyr92	Glu93	Leu94	Lys95	Leu96	Ile97	His98	His99
	Asp104	Pro105	Gly106	Gln107	Ala108	Thr109	Lys110	Tyr111
	Leu112	Tyr113	Glu114	Leu115	Leu116	Try117	Ile124	Leu125
	Met126	Ser130	Ser131	Val132	Ser133	Thr134	Leu135	Val136
	Ala137	Glu138	Ala139	Ala140	Arg141	Met142	Val147	Leu148
	Ser149	Pro155	Ala156	Leu157	Ser158	Phe166	Arg168	Ser326
	Gln327	Glu328	Phe329	Val330	Glu331	Lys332	Leu333	Thr334
	Lys335	Glu349	Ala350	Pro351	Leu352	Ala353	Tyr354	Asp355
	Ala356	Ile357	Trp358	Ala359	Leu360	Ala361	Leu362	Ala363
	Leu364	Asn365	Lys366	Thr367	Ser368	Gln386	Thr387	Ile388
	Thr389	Asp390	Gln391	Ile392	Tyr393	Arg394	Ala395	Met396
	Asn397	Phe401	Glu402	Gly403	Val404	Ser405	Gly406	His407
	Val408							
Lobe 2: Alignment								
**Residue**	Lys144	Ile145	Ala146	Thr147	Ile148	Gln196	Glu219	Ile220
	Thr221	Phe222	Arg223	Gln224	Ile244	Ile245	Val246	Gly247
	Leu248	Val272	Trp273	Phe274	Leu275	Ile306	Thr307	Thr308
	Glu309	Thr420	Leu421	Ile422	Glu423	Gln424	Lys432	Ile433
	Gly434	Tyr435						

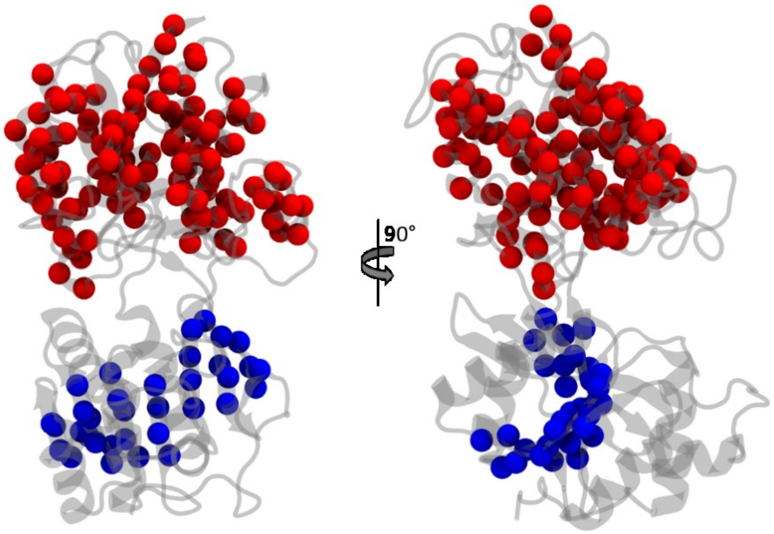

Figure S1. An illustration of the selected Cα atoms used for alignment (blue spheres) and measurement (red spheres). The red spheres illustrate Cα atoms of lobe 1 and blue spheres Cα atoms located in lobe 2.

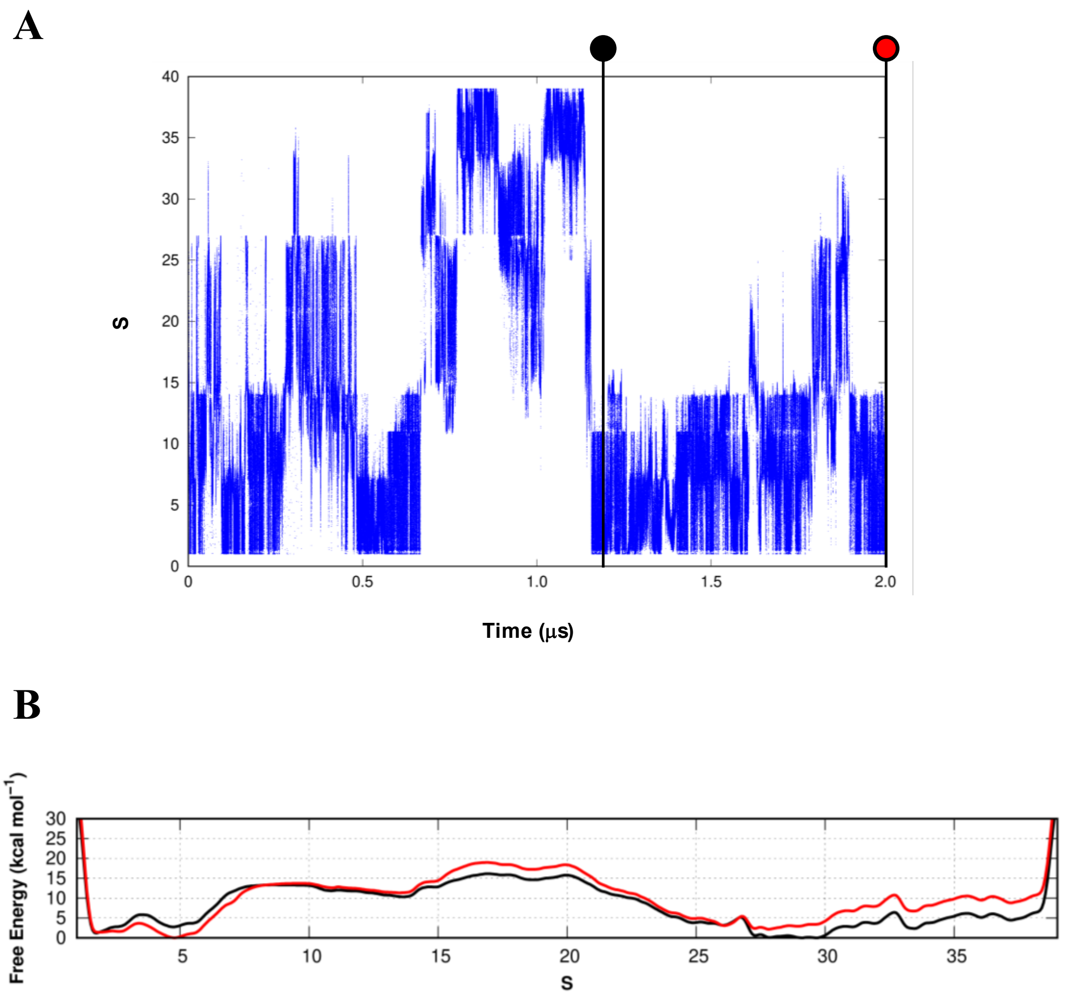

Figure S2. A) Evolution of the *S* variable during the course of the metadynamics simulation. B) Comparison of the 1D-free energy profiles calculated after the main recrossing event (black curve) and at the end of 2 μs metadynamics simulation (red cruve). The corresponding states used for reconstructing the free energy are also shown in panel A).

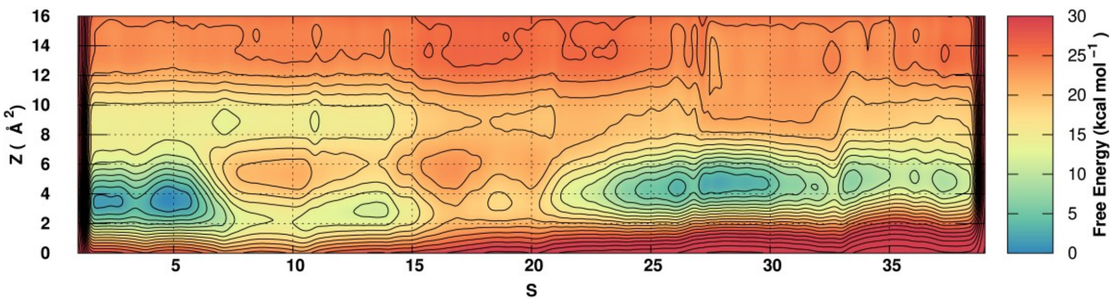

Figure S3. 2D-free energy evaluated at the end of 2 μs of metadynamics sampling.

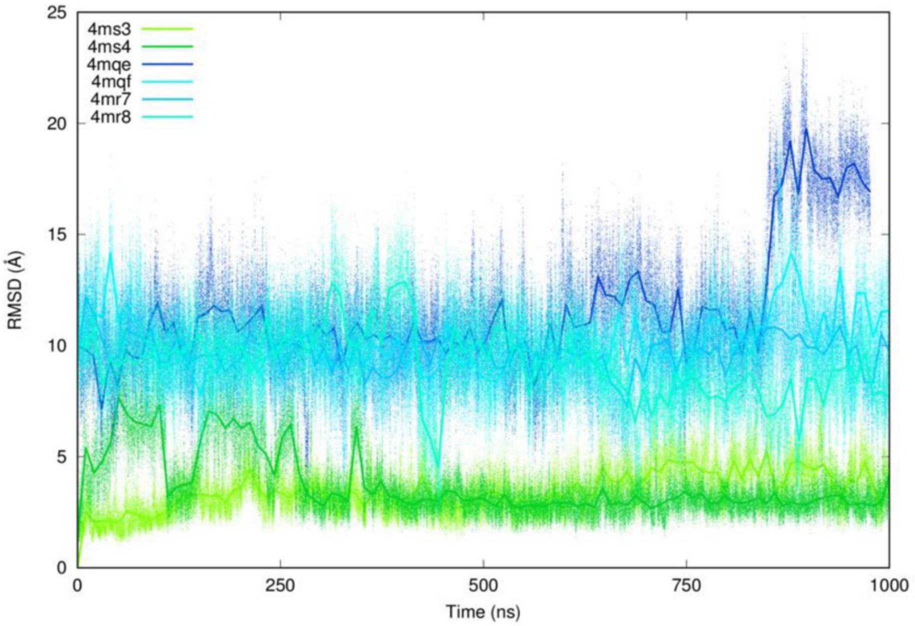

Figure S4. RMSD (Å) of the Cα against time (ns) of six 1 μs-long MD simulations initialized from distinct GABA$_B$-R VFT X-ray structures, using an active/closed structure (PDB ID 4MS3) as a reference. Two structures were initially in the active/closed (green) and four structures were initially in the inactive/open conformation (blue). The darker lines represent the running average for each structure.

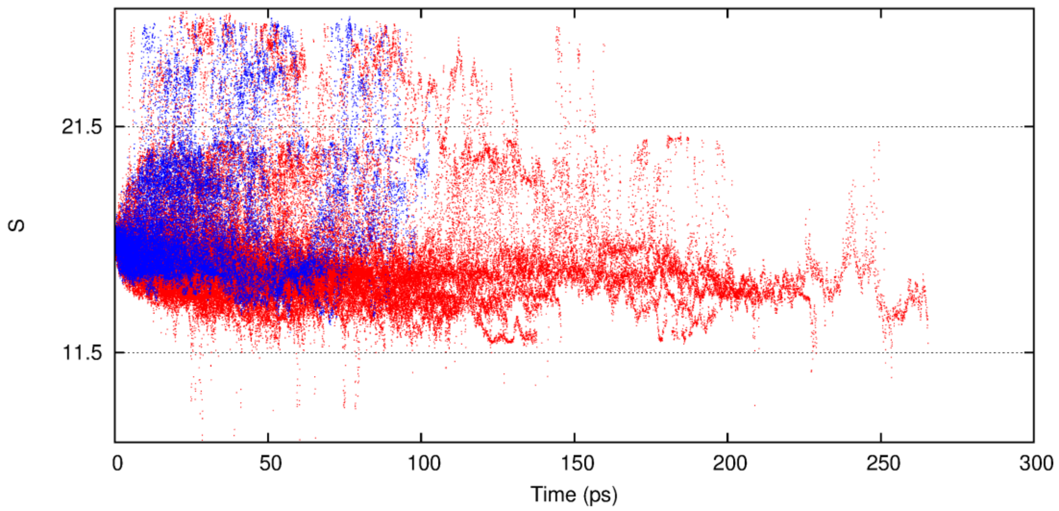

Figure S5. Committor analysis. Evolution of the S variable in time evaluated for multiple simulations started in close proximity of the transition state. Trajectories committed to basin A ($S \leq 11.5$ and $Z \leq 0.05$ nm^2) and B ($S \geq 21.5$ and $Z \leq 0.05$ nm^2) are shown in red and blue, respectively.
